# MAE-TransRNet: An improved transformer-ConvNet architecture with masked autoencoder for cardiac MRI registration

**DOI:** 10.3389/fmed.2023.1114571

**Published:** 2023-03-09

**Authors:** Xin Xiao, Suyu Dong, Yang Yu, Yan Li, Guangyuan Yang, Zhaowen Qiu

**Affiliations:** ^1^College of Information and Computer Engineering, Northeast Forestry University, Harbin, China; ^2^Department of Cardiovascular Surgery, Beijing Anzhen Hospital, Capital Medical University, Beijing, China; ^3^First Affiliated Hospital, Jiamusi University, Jiamusi, China

**Keywords:** deformable image registration, vision transformer, masked autoencoder, self-supervised learning, multi-head self-attention

## Abstract

The heart is a relatively complex non-rigid motion organ in the human body. Quantitative motion analysis of the heart takes on a critical significance to help doctors with accurate diagnosis and treatment. Moreover, cardiovascular magnetic resonance imaging (CMRI) can be used to perform a more detailed quantitative analysis evaluation for cardiac diagnosis. Deformable image registration (DIR) has become a vital task in biomedical image analysis since tissue structures have variability in medical images. Recently, the model based on masked autoencoder (MAE) has recently been shown to be effective in computer vision tasks. Vision Transformer has the context aggregation ability to restore the semantic information in the original image regions by using a low proportion of visible image patches to predict the masked image patches. A novel Transformer-ConvNet architecture is proposed in this study based on MAE for medical image registration. The core of the Transformer is designed as a masked autoencoder (MAE) and a lightweight decoder structure, and feature extraction before the downstream registration task is transformed into the self-supervised learning task. This study also rethinks the calculation method of the multi-head self-attention mechanism in the Transformer encoder. We improve the query-key-value-based dot product attention by introducing both depthwise separable convolution (DWSC) and squeeze and excitation (SE) modules into the self-attention module to reduce the amount of parameter computation to highlight image details and maintain high spatial resolution image features. In addition, concurrent spatial and channel squeeze and excitation (scSE) module is embedded into the CNN structure, which also proves to be effective for extracting robust feature representations. The proposed method, called MAE-TransRNet, has better generalization. The proposed model is evaluated on the cardiac short-axis public dataset (with images and labels) at the 2017 Automated Cardiac Diagnosis Challenge (ACDC). The relevant qualitative and quantitative results (e.g., dice performance and Hausdorff distance) suggest that the proposed model can achieve superior results over those achieved by the state-of-the-art methods, thus proving that MAE and improved self-attention are more effective and promising for medical image registration tasks. Codes and models are available at https://github.com/XinXiao101/MAE-TransRNet.

## 1. Introduction

Medical image registration has been considered a vital analytical task in medical image processing, especially for the registration of deformable non-rigid organs. It is capable of providing doctors with a wide variety of complementary information regarding lesions (1). The systole and diastole of the heart chambers play a vital role in maintaining the ejection function of the heart. Certain heart diseases can lead to changes in the shape of the ventricles, thus resulting in abnormal motion. For instance, hypertrophic cardiomyopathy may cause the localized thinning of the ventricular wall. Inadequate aortic valve closure can cause lesions (e.g., enlarged ventricular chambers). Thus, the study on cardiac registration takes on a critical significance in quantifying cardiac motion, which helps doctors predict the progression of patients diseases in future and conduct precise medical treatment. Moreover, cardiovascular magnetic resonance imaging (CMRI) presents accurate morphological information and a better soft tissue contrast ratio of the human heart (2), which contributes to the diagnosis of a wide variety of cardiac abnormalities. CMRI has become the gold standard in the analysis of cardiac motor function, viability, and abnormalities.

The registration of cardiac images is considered a complex task, which is primarily indicated by two aspects:

(1) Non-rigid and complex motion. The heart undergoes very complex motion and deformation in the cardiac cycle. In addition to the well-known overall deformation (e.g., expansion or contraction), the heart also undergoes overall rigid motion and local deformation, thus making it have a more complex non-rigid periodic motion than other soft tissues (3). Furthermore, due to this motion, the morphology of the slices of the heart varies significantly within continuous time frames of a cardiac cycle, thus making accurate tracking of cardiac motion a difficult task.

(2) Scarcity of anatomical landmarks. There are fewer precise anatomical landmarks required to characterize cardiac motion than to resize other soft tissue structures. Moreover, the labels are more difficult to obtain. Notably, the lack of reliable identifiable landmarks in the myocardial wall makes it difficult for registration (4).

The registration of cardiac images is significantly more complicated than that of other tissues and organs' images due to the aforementioned two major problems.

However, with the rise of deep learning technology over the past few years, traditional registration methods with low accuracy, complex and tedious iterative processes, and high time costs have been unable to reduce the difficulties of today's medical image registration. Thus, deep learning methods based on deep neural networks have become the key to solving the bottleneck of medical image registration performance (5–7). Different training methods are largely divided into three types, namely, supervised learning, unsupervised learning, and weakly supervised learning. In existing research, Rohe et al. (8) proposed SVF-Net, a fully convolutional network based on the U-Net structure. This network replaces all layers in the conventional U-Net (9) network with convolutional layers. In addition, the model combines global semantic information from the deep network and local positional information from the shallow network, and it predicts the SVF 3D velocity field using ROI from the segmentation to supervise 3D cardiac image registration. Unsupervised learning methods have been a research hotspot in the field of registration since there have been rare labels related to cardiac tissue motion analysis. Krebs et al. (10) proposed a low-dimensional multiscale probabilistic deformation network based on conditional variational autoencoder (CVAE). This network is capable of learning from unlabeled cardiac data, which can be used for the registration of deformable soft tissue structures (e.g., heart and brain). Balakrishnan et al. (11) optimized a simple U-Net network, named VoxelMorph, which can be trained in an unsupervised or supervised manner to achieve MRI registration results by defining a loss function consisting of a mean square error (MSE). The loss function comprises a similarity measure and a smoothing constraint on the deformation field. Some researchers, inspired by the above-unsupervised methods, also proposed a weakly supervised strategy to solve the problem of sparse anatomical signatures of tissues and organs. Hu et al. (12) proposed a method to infer the registration field parameters from the high-level information contained in a small number of existing anatomical labels. These researchers introduced existing annotations in the region around the target at the training stage to introduce additional information for optimizing the network parameters and increasing the registration accuracy. Deep learning based on medical image registration methods, especially using convolutional neural networks, have shown more significant improvements in registration performance over the past few years. Increasing methods have been proposed to solve the problems of slow computation and less information captured using existing 2D/3D registration methods (13). However, the current mainstream frameworks primarily use convolutional neural networks as the backbone, and the conventional convolutional operation is to extract features by sliding a window with a convolutional kernel size. Moreover, the perceptual field is limited to a fixed-size region, which is only effective in extracting local features and has some limitations in acquiring global information (14). The Transformer, originally applied in the field of NLP, has gradually become a novel alternative architecture for extracting global features in recent years since it is effective in capturing long-range global location information (15). Nevertheless, since the Transformer is insufficient to extract local detailed features, relevant research has emerged to fuse the advantages of Transformer in extracting global information and CNN in extracting local information to complement each other. Vision transformer (16) is capable of dividing the image data into patches and then interpreting these patches as sequences to take them as input. The above tokens are handed it over to the Transformer encoder for processing. Thus, Chen et al. (17) first proposed a hybrid model of Transformer and CNN (TransUnet), thus preserving the U-shaped structure of U-Net and introducing the Transformer encoder structure. The input image is first passed through a series of convolution operations to generate feature maps of different resolutions. In addition, the network serializes the feature maps output from the last layer as the patches. These patches are input to the Transformer layer for encoding. Subsequently, a feature sequence with self-attentive weights is obtained through Transformers encoding, and it is reshaped to the image size and then upsampled, which is combined with different high-resolution CNN features derived from the encoding path in the upsampling process to achieve a more accurate medical image segmentation tasks. Chen et al. (18) proposed ViT-V-Net fusing the basic registration framework-VoxelMorph based on the V-Net (19) structure with the vision transformer-based encoder to fully use the spatial correspondence obtained from the 3D volume for more accurate registration. As a result, the network can be better in extracting registration field features and extracting global features. The network is capable of extracting global features, while preserving as many local features as possible between image contexts.

Although introducing the Transformer has been very effective in solving problems (e.g., the loss of deep local feature information), numerous Transformer baselines and hybrid models have been proposed to solve the above problems. In fact, the Transformer is transferred from the NLP to the CV field, and a relatively large gap exists between the above two fields in understanding images and texts. Compared with the high information density of linguistic text information, the image information is highly redundant, thus making it relatively difficult for the model to predict the information density. In addition, considerable information irrelevant to the task objective may be included in the scope of the model learning, so the model should spend a lot of parameter capacity in learning. Moreover, a significant gap exists in the design of Transformer-based structures for NLP tasks and CV tasks. Decoding linguistic information may be easier than images, and reconstructing pixels is more complex than reconstructing words, so the design of the Transformer's internal structure is significantly correlated with the learning effect of implicit semantic representation during image decoding. Due to the above analysis and the emergence of the problem, He et al. (20) transferred the method with masked operation from NLP to the CV field. They developed a relatively simple strategy, i.e., randomly masking a certain percentage of the image patches, so the model can learn more useful features and can predict the information of the missing pixels. This architecture is capable of effectively achieving good results in classification, segmentation, and detection tasks.

In the meantime, the self-attention mechanism plays a crucial role in Transformer encoders, and its variants have been used to varying degrees in text, image, speech, and video tasks (21–23). The self-attention mechanism can filter out the features which are useful for the target task, and improve the model computation efficiency to a certain extent. It can pay more attention to the feature correlation between the data, to solve the problems of network information redundancy, gradient dispersion, and the difficulty of handling variable-length sequences. The current multi-head self-attention mechanism used in the traditional vision transformer maps each sequence into three different feature spaces (Q, K, V), and then calculates the attention weights by scaled dot product, which selects parallel multiple features from the input features for fusion. The attention mechanism based on the scaled dot product can capture the global contextual information of the feature sequence. However, in terms of computational complexity, assuming that the sequence length is set to N of dimension D, the dot product computation is essentially a multiplication between a matrix of dimension *N*×*D* and a matrix of *D*×*N* with a time complexity of *O*(*n*^2^*d*). In natural language processing tasks and some speech recognition tasks, many related studies have simplified the computation of the self-attention mechanism. It is necessary to consider some strategies to make it better for vision tasks and to reduce the computational complexity of self-attention.

Inspired by their research, we propose a novel Transformer-ConvNet model (MAE-TransRNet) using the MAE's strategy for cardiac MRI registration.

This study aims to enhance the performance of cardiac MRI registration by combining the advantages of CNN and Transformer. In this study, the transformer structure, which is currently popular, is primarily adopted to fuse the basic structure of the existing unsupervised registration baseline-VoxelMorph. We also explore the effect of the improved self-attention mechanism on the effect of feature aggregation. In addition, the attention mechanism and the superiority of the currently proposed Transformer structure with a MAE in increasing the registration accuracy of 3D medical images are investigated. The main contributions of this study are summarized into the following aspects:

(1) We propose a new hybrid multi-head self-attention module (HyMHSA) for vision tasks. The original query-key-value-based dot product computation unit is replaced with a dense synthesis unit that directly computes the attention weights. Meanwhile, the attention module restricts the interactions between sequences by exploiting the correlation between adjacent contexts of sequences, which makes the attention weights interact only between a portion of adjacent tokens and fuses them with the dot product form of the computation unit to reduce the computational burden.

(2) We introduce the concurrent spatial and channel squeeze and excitation (scSE) module (24) in the CNN's downsampling structure. In the Transformer encoder, squeeze and excitation module (25) is introduced after the attention to the Transformer structure, so as to reduce the feature redundancy in the self-attention mechanism in the ViT model, while increasing the richness of the cardiac image features.

(3) The structure of the conventional ViT model is improved based on Masked Autoencoder (MAE) (20). The application of the Transformer combined with VoxelMorph is deeply considered in medical image registration based on the existing research, and the Transformer is employed as a baseline to make corresponding model improvements. The proposed model is named MAE-TransRNet.

## 2. Related work

### 2.1. Deformable image registration baseline–VoxelMorph

Convolutional neural networks have progressively replaced the conventional registration methods based on mutual information with the development of deep learning in recent years. VoxelMorph (11) was proposed in 2019 and has been extensively used as a baseline in medical image registration. The VoxelMorph framework can learn registration field parameters from 3D volumetric data, and the encoder-decoder structure based on U-Net (9) structure is adopted to combine shallow features and deep features and reduce the information loss of features. Moreover, VoxelMorph provides two training strategies. One training strategy is based on the grayscale value of the image to make the similarity to maximize the similarity loss and smoothing loss. This part is primarily pure unsupervised learning method for iterative optimization. The other training strategy introduces additional segmentation labels of the image as the auxiliary information based on the unsupervised method by obtaining the dice performance between the image pairs of segmentation labels at the training stage, thus increasing the registration effect. In this study, the superiority of the VoxelMorph baseline framework in the medical image registration is considered, and the skip connection structure of the VoxelMorph model architecture is redesigned and transferred into a long-range skip connection structure containing CNN encoder and decoder. This design is capable of combining the local information of feature maps at different scales more effectively and increasing the feature extraction capability.

### 2.2. Multi-head self-attention in transformer encoder

The multi-head self-attention module selects multiple pieces of information from the inputs and learns feature representations from different representation subspaces at different locations. The operation of multi-head attention can be described as mapping a query and a set of key-value pairs to the output, where the query, key, and value are denoted by Q, K, and V. Then, the three-part linear mapping is input to the attention mechanism based on scaled dot product to perform *h* attentions in parallel computation (*h* refers to multiple heads). The formula for each dot product attention computation is as follows:


(1) $$\begin{array}{l} Attention\left(Q,K,V\right)=softmax\left(\frac{QK^{T}}{\sqrt{D_{k}}}\right)V \end{array}$$

$\frac{1}{\sqrt{D_{k}}}$ is the attention deflator that mitigates the gradient disappearance. Then the results of h-heads scaled dot product attention are concatenated to obtain the final multi-head attention output feature vector:


(2) $$\begin{array}{l} MultiHead\left(Q,K,V\right)=Concat\left(head_{1},...,head_{h}\right)W^{O} \end{array}$$

(3) $$\begin{array}{l} where\;head_{i}=Attention\left(QW_{i}^{Q},KW_{i}^{K},VW_{i}^{V}\right) \end{array}$$

**Figure 1:**
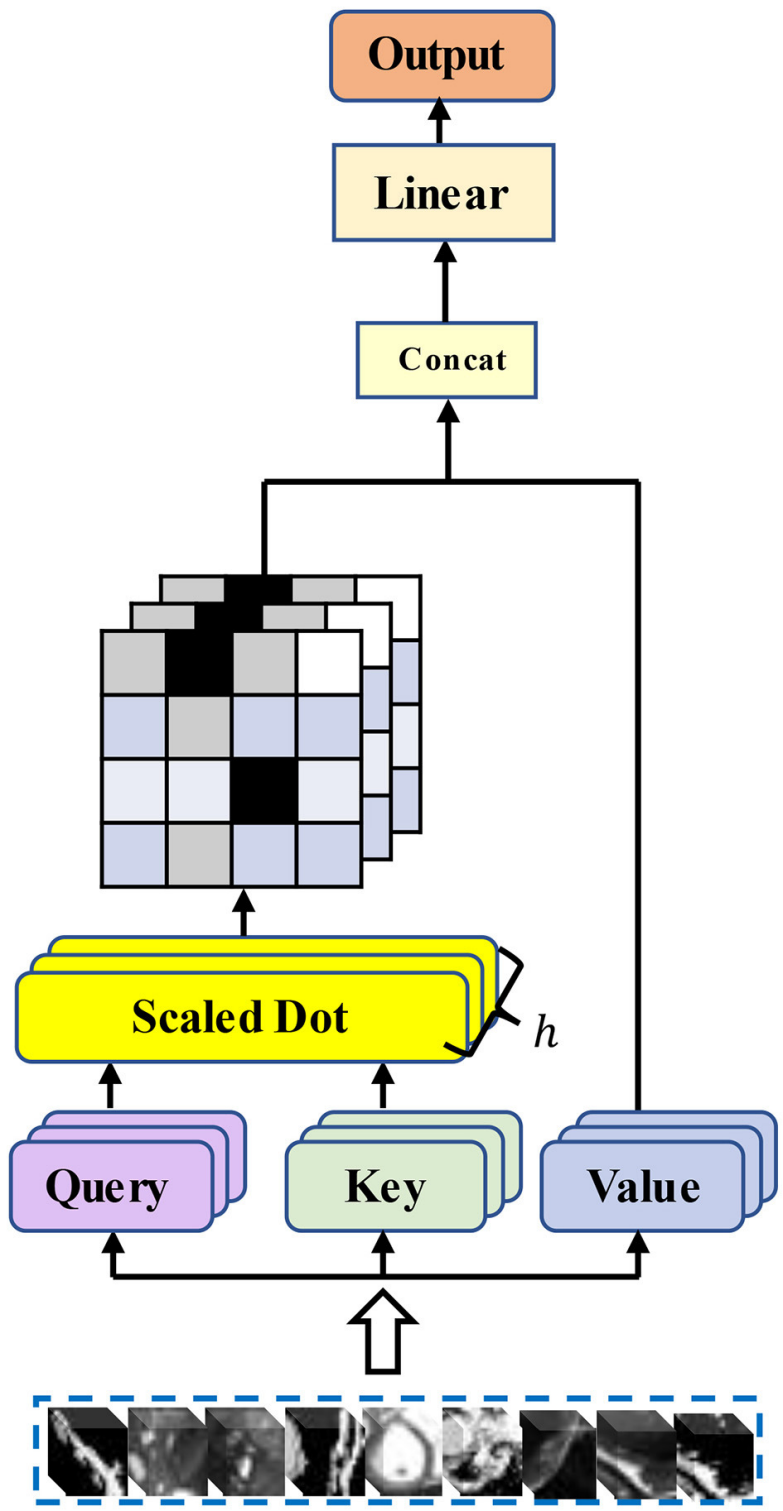
Structure of the traditional multi-head self-attention mechanism.

*W*^*Q*^, *W*^*K*^, and *W*^*V*^ denote the weight parameter matrixes corresponding to Q, K, and V. Figure 1 illustrates the structure of the traditional multi-head self-attention mechanism.

Self-attention models have been widely used in various fields. For query, key, and value, sequences of three vectors generated by tokens through linear layers, a considerable number of researchers have considered how to reduce the computation of attention. Quadratic, for a matrix with *N*×*N*, we may not need the value of each position on the matrix to participate in the attention computation. Furthermore, for the global context information of the Transformer, we do not need to consider all the information from the beginning to the end. A sequence obtained from the cut image patches has a very long length. That is, when calculating the value of the current position, only its neighboring positions are considered. The range of neighboring positions to consider, the choice of position, and the choice of the sequence length to be calculated are all important factors that currently affect the complexity of attention computation in the vision domain. Chiu and Raffel (26) introduce a scalable and variable sliding window for attention computation, and Tay et al. (27) abandon the query-key-based interactive attention weight learning approach and propose a dense synthesizer that uses two feed-forward linear layers to predict the attention weight parameters. Xu et al. (28) further proposed a local dense synthesizer. They restrict the attention computation to a local area around the current central frame. However, improved works based on the self-attention mechanism are rarely found in medical image registration tasks. Our work is a new attempt. We introduce an improved self-attention mechanism into our Transformer encoder and explore whether the attention module applicable to text, as well as speech tasks, can be well applied to our registration tasks.

### 2.3. Squeeze and excitation block in feature extraction

The convolutional operation is the core of conventional convolutional neural networks, which are based on local perceptual fields to fuse features in spatial and channel dimensions. The squeeze and excitation block proposed by Hu et al. (25) in 2018 places a focus on the research relating to the channel dimension and explores the feature relationships between channels, which can adaptively adjust the features on the channel dimension. The squeeze and excitation block can be stacked in many classical classification network structures (e.g., AlexNet and ResNet), and it has high performance on datasets (e.g., ImageNet). Inspired by the squeeze and excitation module, Guha Roy et al. (24) explored a fusion module combining channel dimension features and spatial dimension features to “reconstruct” features both in space and channel. Thus, the network can focus more on learning features that are more significance in downstream tasks, and it exhibits high applicability in 2D and 3D scenes. For the common tasks in current medical image analysis, (e.g., segmentation and registration), more insights should be gained into the spatial information at the pixel level of the image. Now, the embedding structure of such modules has been extensively used in the field of medical images (e.g., brain MRI and enhanced CT's segmentation tasks). Based on the above research, squeeze and excitation module and concurrent spatial and channel squeeze and excitation (scSE) module are embedded into the proposed model, and the role of the above two modules in improving the performance of the registration model is explored. The importance of different levels of features is adjusted, so the model can learn more valuable high-level features, and features that are less important for the target task are given less attention. Thus, richer spatial and channel information can be obtained at the pixel level. The relative importance of attention in both dimensions is calibrated simultaneously, which leads to further accuracy improvements in downstream registration tasks.

### 2.4. Transformers in vision and self-supervised learning

With the prevalence of Transformer architectures migrated from the NLP domain, increasing variants of Transformer-ConvNet have high performance in computer vision tasks. Transformer structures are now extensively employed in vital tasks (e.g., medical image segmentation, medical image registration, and reconstruction) because of their superiority in capturing global contextual information and the localization of CNN convolutional operations for fine feature extraction. The TransUnet proposed by Chen et al. (17) is the first attempt at the Transformer-ConvNet structure, and it has achieved effective results in the segmentation tasks of cardiac and abdominal multiple organs. Several important works have also emerged in registration tasks, suggesting that the splicing and Transformer-ConvNet structures can effectively consider the advantages of both in their respective fields. However, with the emergence of some relevant in-depth studies, several problems are caused as follows:

(1) Numerous studies have suggested that the critical factor for learning efficiency is the scale of dataset, besides some problems of the model. The ability to learn valid information from considerable unlabeled data has been a crucial research topic in medical image analysis tasks. The number of data required to train the vision Transformer is significantly higher than that of a conventional convolutional neural network, especially the standard dataset with annotations. However, for medical images with a small sample size, it is undoubtedly challenging to obtain many labeled datasets, and the problem of data starvation always exists in the research on the vision Transformer architecture.

(2) The Transformer structures adopted to fuse CNNs are primarily migrated versions of structures based on NLP tasks, and the information density contained in the text is significantly different from the images. The features extracted by the Transformer encoder may be too complete and contain some redundant information, so it is imperative to remove redundancy.

In medical images, the anatomical structure of the respective organ has a certain correlation between different contextual slice information, and it is also correlated with the features of the neighboring regions around the target region. The learning of the neighboring information and contextual information between pixels can facilitate the representation of advanced features. With the continuous development of self-supervised learning, the Transformer structure combined with the self-attentive mechanism (29) can break through the state of the art continuously. Self-supervised learning essentially provides a reliable learning path that allows the network to learn from large amounts of unlabeled data to be more capable of feature extraction. In fact, self-supervised learning is divided into several processes. (1) First, the basic structure or characteristics of the large amount of unlabeled data (which can be interpreted as built-in prior knowledge) are employed. Together with the relevant requirements of the task definition, some certain properties of the data are adopted instead of manual labeling, which can be interpreted as generating pseudo-labels for the images and initially training the network. Thus, it can extract features, i.e., the initial learning ability. (2) Second, the network is fine-tuned with a small amount of labeled data, so the network can further satisfy other tasks such as classification, segmentation, and registration.

The Transformer refers to an encoder -decoder integration based purely on an attention mechanism. In the current vision tasks, more novel strategies are urgently required to help models learn image features with powerful representations due to the different natures of visual information and textual information. Moreover, the MAE recently proposed by Kaiming He et al., has been well-adapted to the vision transformer and has achieved better results in tasks (e.g., classification). We consider that masked autoencoder can be effective in computer vision tasks by destroying most of the patches of the image data and forcing the model to adapt to this defective feature structure when learning the image representation, which significantly reduces the redundancy of the image and creates a more challenging assignment. Finally, the model is enabled to learn the essential features of the image, so a powerful representation of the whole image data is obtained. The design of an asymmetric encoder -decoder structure saves model overhead, in which the encoder accounts for learning high-level feature representations by learning only the visible, unobscured patches, and the obscured patches are represented by a set of shared, learnable latent vectors. Self-supervised learning is further introduced into the visual transformer based on existing research, and the self-encoder with mask operations is applied to the heart image registration task, which can effectively solve the problem of sparsely labeled data and large information density differences between images and texts. Furthermore, applying the expandable MAE to our task and increasing the feature learning difficulty can instead lead to a stronger learning capability of the model.

## 3. Proposed method

### 3.1. Overview

Our MAE-TransRNet is a two-stage registration pipeline. In the first stage (bottom half of the figure), we use masked autoencoder as the encoder for pre-training. The encoder input is a subset of random masking of the image after patch chunking, and a modified self-attention mechanism is used in the Transformer encoder for simplifying the attention weight calculation. It calculates the attention parameter by selecting local contextual location information in the sequence. We reconstruct the complete image with a lightweight Transformer decoder, and the pre-trained model weights contain the powerful global latent features learned by the MAE pre-trained model on the cardiac image. In the second stage (top half of the figure), the pre-trained weights generated in the first stage are passed to the encoder of the registration model, and the registration network is initialized. Our input is 3D cardiac MRI (Ω ∈ *R*^3^), which consists of a single-channel grayscale image of the initial time frame *F* ∈ *R*^*H*×*W*×*L*^ (fixed image) and a single-channel grayscale image of the end time frame *M* ∈ *R*^*H*×*W*×*L*^ (moving image). The proposed model aims to learn the mapping transformations between the image pairs of the initial frame and the final frame. The resolution of the original image is first reduced to a suitable size through the downsampling operation of three convolutional neural networks to obtain a high-level feature representation, and then the spatial features are combined with the channel features by a concurrent spatial and channel squeeze and excitation (scSE) module, and the obtained high-level attention features are fed into the Transformer coding layer with the same structure as the pre-training stage. Going through the CNN decoder, the high-level features are reshaped to the image format. The deformation field is CNN's output, which is applied to the moving image through the warping layer. Here, the model uses the weights learned in the pre-training stage to train the registration network by calculating loss for backpropagation to generate a registration network model with optimal weight parameters. Subsequently, the objective function is minimized, as expressed in Equation (4)


(4) $$\begin{array}{l} \hat{\varphi }=\underset{\varphi }{\text{arg }\min}L\left(F,M,\varphi \right) \end{array}$$

**Figure 2:**
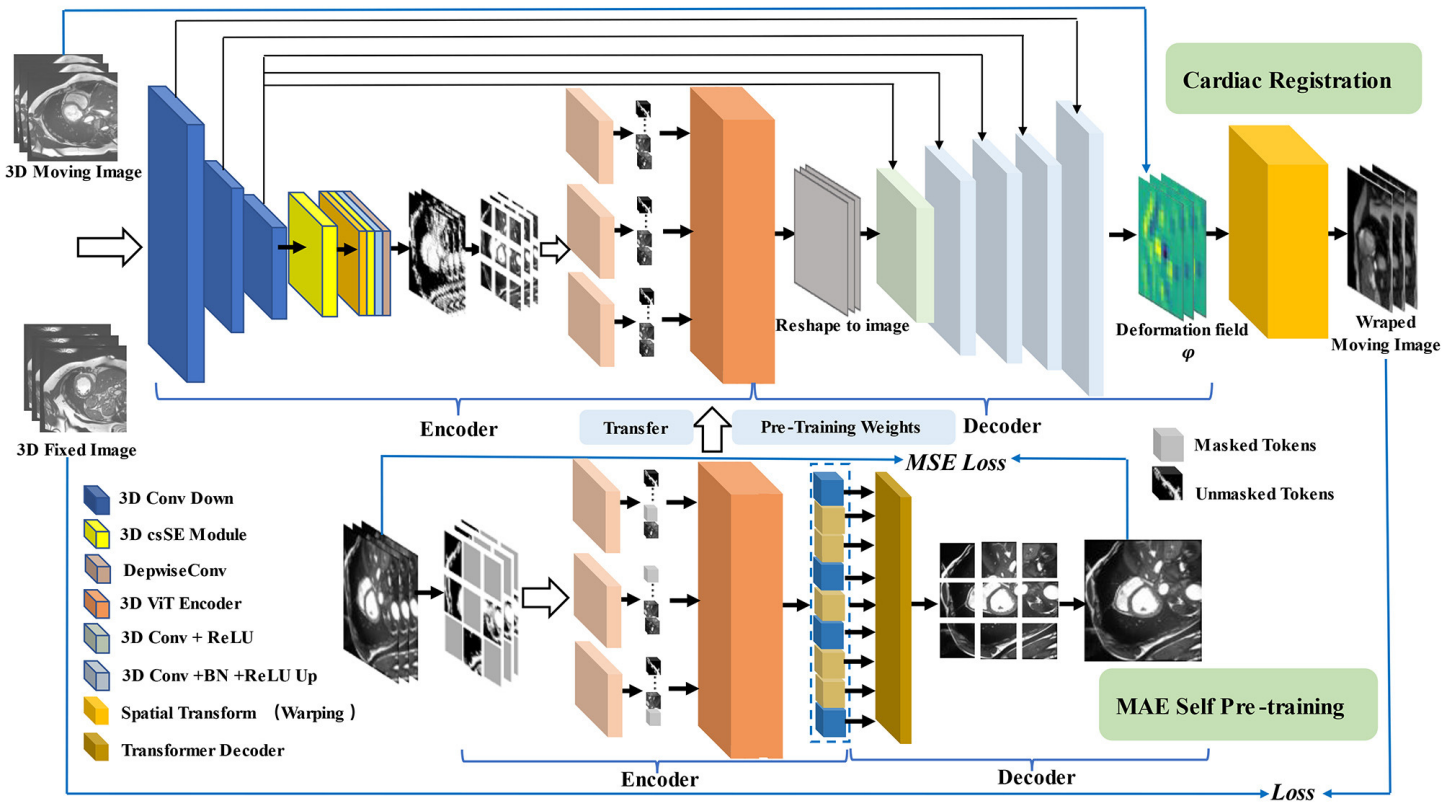
Pipeline for our registration method with MAE Pre-training. In the first stage (shown at the **bottom**), we use multi-branch MAE for pre-training, The input of the Transformer encoder is a subset of unobscured tokens from multiple branches, with a lightweight decoder to reconstruct the complete image. In the second stage (shown at the **top**), the weights obtained in the pre-training stage are used for the initialization of the registration model, the network takes a two-channel 3D volume tensor as our input, which is down-sampled by three convolutions, and then recalibrated with attention weights by scSE module, CNN decoder recovers potential features to image size and up-sample back to the original resolution size, using the spatial transformation function to warp moving image.

φ is obtained as the vector field offset from F to M as a feature of the registration image pair, i.e., φ = *Id*+*u*. *Id* represents the constant transformation operation, and *u* represents the displacement vector field. Figure 2 illustrates the overall pipeline of the proposed method.

### 3.2. Novel multi-head self-attention with SE module

In the Transformer encoder, the core of multi-head self-attention is to map query, key, and value in their respective representation subspaces and merge them back after processing in their respective spaces, which is essentially the decomposition process and re-aggregation of attention features. For the visual domain, each location of each feature mapping contains information about the features at other locations in the same image, which makes the model more adept at capturing the dependencies between features with long spatial intervals. However, in practice, the input of image data is generally high resolution, especially 3D images, which makes the model need to learn longer feature sequences without losing too many fine-grained features of the image, and neither direct processing of the whole image nor downsampling can solve such problems significantly. The presence of inductive bias in CNN structures allows such models to be good at extracting local information. The Transformer structure remains desperate for extensive sample-size medical training data. In the face of such scarce data, we can only achieve this by exploring more powerful feature extractors, introducing some of the properties of inductive bias inherent in CNNs into Transformer, and in particular, embedding efficient convolution modules in the structure of self-attention computation to enhance the attention to small-scale local information in the dataset, which is one of the aims of our study.

We introduce the SE module into the computation of attention. Meanwhile, we embed the depthwise separable convolution (30) into our attention and the feed-forward layer. Given a 3D image as the input *X* ∈ *R*^*B*×*C*×*H*×*W*×*L*^, the input is mapped into three subspaces representing Q, K, and V by a module consisting of deep convolution and point convolution, respectively. The depthwise convolution aggregates the spatial information, and the pointwise convolution aggregates the feature information along the channel dimension. Then we flatten the image features into a long sequence for Transformer encoding by patch embedding and position encoding. SE module is introduced in the respective transformer block to solve the problem that many channels in many current ViT models contain excessive redundant information, as well as to increase the efficiency of the model. After SE modules, the long token (*X* ∈ ℝ^*B*×*N*×*D*^) is compressed into a 1 × 1 × 1 × *D* token, which is equivalent to compressing all global attention features into a high-level feature representation. Moreover, a series of nonlinear mappings are performed for the respective channel of the high-level features. Finally, the weight parameter corresponding to each channel is obtained, and a weight value representing the degree of attention is generated for the respective feature channel. This part aims to learn the nonlinear interaction between each token channel, and the weights are weighted with the original token to obtain the reconstructed attention to the feature representation with shape *B*×*N*×*D*. After the above operation, our input changes from *X* ∈ ℝ^*B*×*C*×*H*×*W*×*L*^ to $X_{Q}/X_{K}/X_{V}\in {\mathbb{R}}^{B\times N\times D}$, formulating as:


(5) $$\begin{array}{l} \begin{array}{c} X_{1}=PoiW\left(DepW\left(X,K_{1}\right),K_{0}\right)\\ X_{2}=PoiW\left(DepW\left(X,K_{2}\right),K_{0}\right)\\ X_{3}=PoiW\left(DepW\left(X,K_{3}\right),K_{0}\right) \end{array} \end{array}$$

(6) $$\begin{array}{l} \begin{array}{c} X_{Q}=SE\left(Patch\text{\_}PosEmd\left(X_{1},H,W,L,C,P\right),r\right)\\ X_{K}=SE\left(Patch\text{\_}PosEmd\left(X_{2},H,W,L,C,P\right),r\right)\\ X_{V}=SE\left(Patch\text{\_}PosEmd\left(X_{3},H,W,L,C,P\right),r\right) \end{array} \end{array}$$

where *DepW* and *PoiW* denote depthwise convolution and pointwise convolution, *K*_0_, *K*_1_, *K*_2_, and *K*_3_ are different kernel sizes, *r* is the reduction ratio of SE module, *X*_*Q*_, *X*_*K*_, and *X*_*V*_ denote the vector representations mapped from the original input to three different subspaces, and *P* denotes the patch size.

The design of the attention module affects the computational efficiency of the vision transformer. Currently, self-attention in vision transformer establishes global long-distance dependencies by interacting information between all regions in the image, which requires neighborhood and global context to achieve. Our approach does not entirely discard the decomposition and aggregation model of multi-headed self-attention while further setting the model's scope to consider neighborhoods. Our hybrid attention reduces the computational effort by restricting the current frame from interacting with its finite neighboring frames. We take one attention head as an example to explain our approach. First, we generate three weight matrixes *W*_1_, *W*_2_, and *W*_3_ for computing attention using the linear layer of SELU. *W*_1_ and *W*_2_ are used to directly generate the attention weights corresponding to *X*_*Q*_ and *X*_*K*_, and *W*_3_ is used to generate the attention weights for “values.” In *W*_2_, we introduce a hyperparameter *cn*, which represents the contextual neighbors. This parameter restricts the contexts around the current location considered in the attention calculation. Thus, the dimension of the original weight calculation is reduced from *N* to *cn*. The model shares attention weights among only a limited number of locally adjacent contexts, significantly reducing time complexity. The input token ($X_{Q}/X_{K}/X_{V}\in {\mathbb{R}}^{B\times N\times D}$) is computed by attention weighting to obtain the query token ($S_{XQ}\in {\mathbb{R}}^{B\times N\times cn}$) with local contextual information and key token ($S_{XK}\in {\mathbb{R}}^{B\times N\times cn}$), value token ($S_{XV}\in {\mathbb{R}}^{B\times N\times D}$) are obtained directly by *W*_3_ weighting. Furthermore, we introduce the variable *j* to compute the weights of each *cn* position above and below the *j*-centered position in the token with local attention, weight it with the value token, and sum it to obtain two vector outputs *AttnQ*_*V*, *AttnQ*_*K* containing local adjacency context information. Since query and key are obtained by locally computing the full dense attention synthesized directly, we call this part the local dense attention computation module. The output of local dense attention is calculated by:


(7) $$\begin{array}{l} \begin{array}{ll} S_{XQ} & =\text{Soft}\max\left(SELU\left(X_{Q}W_{1}\right)W_{2}\right)\\ S_{XK} & =\text{Soft}\max\left(SELU\left(X_{K}W_{1}\right)W_{2}\right)\\ S_{XV} & =X_{V}W_{3} \end{array} \end{array}$$

(8) $$\begin{array}{l} \begin{array}{c} Out_{Q}^{n}=\overset{cn-1}{\underset{j=0}{\sum }}S_{XQ}^{n,j}S_{XV}^{n+j-\left\lfloor \frac{cn}{2}\right\rfloor }\\ Out_{K}^{n}=\overset{cn-1}{\underset{j=0}{\sum }}S_{XK}^{n,j}S_{XV}^{n+j-\left\lfloor \frac{cn}{2}\right\rfloor } \end{array} \end{array}$$

(9) $$\begin{array}{l} \begin{array}{c} AttnQ\text{\_}V=Out_{Q}^{n}W_{3}\\ AttnK\text{\_}V=Out_{K}^{n}W_{3} \end{array} \end{array}$$

where $W_{1}\in {\mathbb{R}}^{D\times D}$, $W_{2}\in {\mathbb{R}}^{D\times cn}$, and $W_{1}\in {\mathbb{R}}^{D\times D}$ are three learnable weights and *n* denotes the number of tokens.

Finally, we aggregate the attention of the three components Q, K, and V by the traditional multi-head self-attention computation module to obtain the feature representation of hybrid attention in one attention head, and then we concatenate the outputs of all the *h* heads and calculate the output of the HyMHSA block, formulating as:


(10) $$\begin{array}{l} AttnOut=MHSA\left(AttnQ\text{\_}V\cdot W^{Q},AttnQ\text{\_}K\cdot W^{K},S_{XV}\cdot W^{V}\right) \end{array}$$

(11) $$\begin{array}{l} HyMHSA\left(X\right)=Concat\left(AttnOut_{1},...,AttnOut_{h}\right)W^{m} \end{array}$$

**Figure 3:**
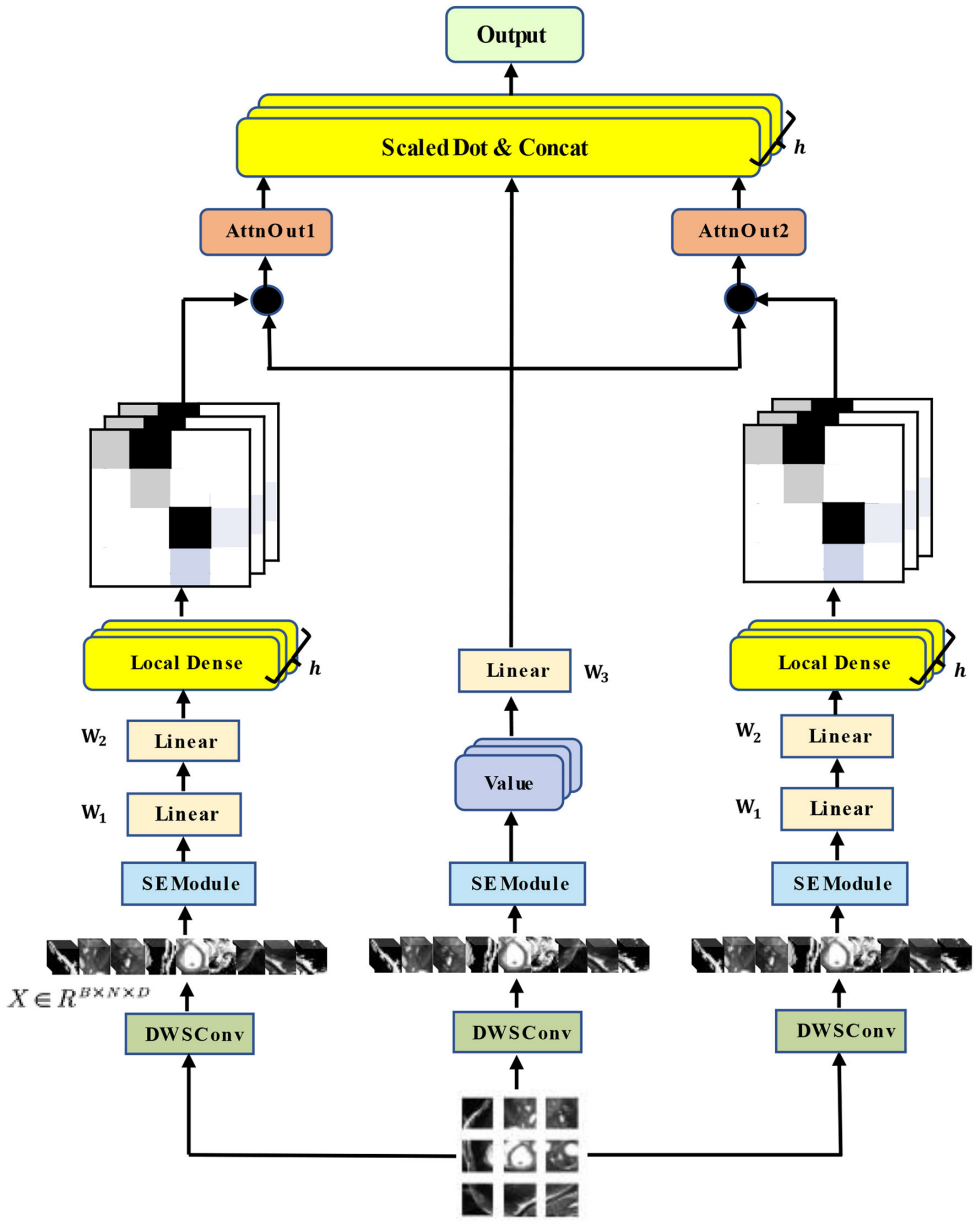
Architecture of our HyMHSA module. It is a hybrid version of the traditional multi-head self-attention mechanism and local dense self-attention mechanism.

Our architecture of the hybrid multi-head self-attention mechanism is shown in Figure 3.

### 3.3. Squeeze and excitation module in 3D CNN encoder

**Figure 4:**
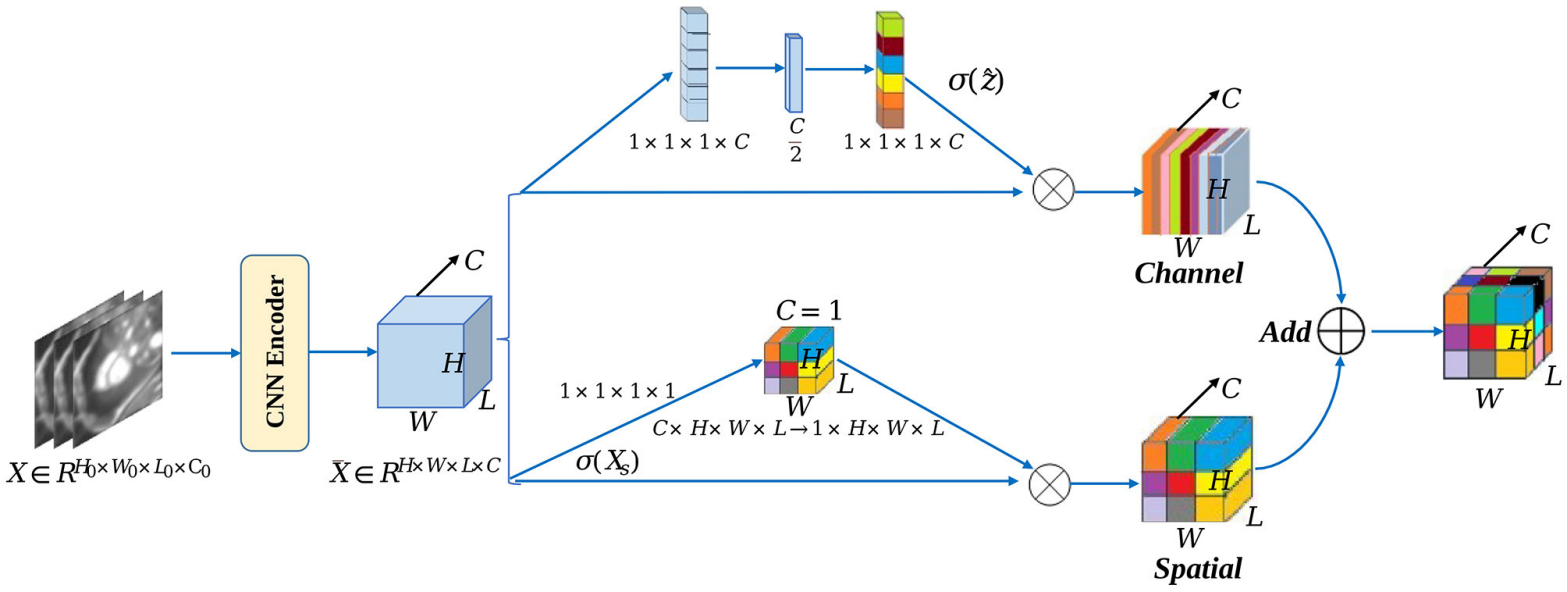
scSE module in CNN encoder.

The channel and spatial dimensional parallel attention mechanism modules are introduced in the CNN encoder before the Transformer structure to operate on convolutional features using a dual-dimensional parallel extraction of attention features, with input feature maps of $\bar{X}\in R^{H\times W\times L\times C}$. Moreover, the attention mechanism modules [e.g., spatial squeeze and channel excitation block (cSE) and channel squeeze and spatial excitation block (sSE)] are applied to 3D CNN (Figure 4) The spatial attention module consists of a global average pooling layer and a fully connected, ReLU activation layer (ẑ = *W*_1_(δ(*W*_2_*z*))) behind it. This module generates the intermediate feature vector *z* ∈ *R*^1 × 1 × 1 × *C*^ *via* the pooling layer while generating *n*-th element, which is expressed as follows:


(12) $$\begin{array}{l} z_{n}=\frac{1}{H\times W\times L}\overset{H}{\underset{i}{\sum }}\overset{W}{\underset{j}{\sum }}\overset{L}{\underset{k}{\sum }}{\text{u}}_{n}\left(i,j,k\right) \end{array}$$

In this step, the global spatial information of the image features is also embedded into the feature vector *z*. With the variation of the squeeze and excitation module, the entire attention recalibration process is expressed as follows:


(13) $$\begin{array}{l} {\bar{X}}_{cSE}=F_{cSE}\left(\bar{X}\right)=\left[\sigma \left(\hat{z_{1}}\right)x_{1},\sigma \left(\hat{z_{2}}\right)x_{2},\cdots \;,\sigma \left(\hat{z_{c}}\right)x_{c}\right] \end{array}$$

where *c* denotes the attention weight of each channel, emphasizing high-importance features and suppressing low-importance features, assigning different levels of importance to the respective channel. The other part targets the fine-grained pixel information in cardiac MRI. This part is enabled us to deeply mine the channel information of the feature map and then spatially excite it. The feature vector is expressed as $\bar{X}=\left[x^{1,1,1},x^{1,1,2},\cdots \;,x^{i,j,k},\cdots \;,x^{H,W,L}\right]$, and the linear representation of the feature projection ($X_{s}=W_{sq}\cdot \bar{X}$) is obtained through convolution operation. The attention recalibration process is illustrated as follows:


(14) $$\begin{array}{l} {\bar{X}}_{sSE}=F_{sSE}\left(\bar{X}\right)=[\sigma \left(X_{s\left(1,1,1\right)}\right)x^{1,1,1},\sigma \left(X_{s\left(i,j,k\right)}\right)x^{i,j,k},\cdots ,\\ \;\sigma \left(X_{s\left(H,W,L\right)}\right)x^{H,W,L}] \end{array}$$

The value of each σ represents the relative importance of the spatial information (*i, j, k, c*) for a given feature map. Accordingly, the combination of the two modules allows features on channel and spatial aspects to be considered more often in the learning process of the network. The formula is:


(15) $$\begin{array}{l} {\bar{\text{X}}}_{scSE}={\bar{\text{X}}}_{cSE}+{\bar{\text{X}}}_{sSE} \end{array}$$

### 3.4. 3D vision transformer with MAE as deformable registration core architecture

#### 3.4.1. 3D vision transformer architecture

The conventional 3D ViT architecture is borrowed as the backbone for pre-training and downstream registration tasks, and the feature maps containing some high-level feature information obtained from three downsampling operations are employed as the input of ViT: $\bar{X}\in {\mathbb{R}}^{H\times W\times L\times C}$. The size of *P*×*P*×*P* non-overlapping patches is adopted to slice the high-dimensional images to get $N=\frac{HWL}{P^{3}}$ patches. These patches are flattened into *P*^3^*C*-dimension vectors, and the serialized representation with high-level features is obtained. To preserve the position information, position embedding is introduced after patch embedding, and the vector of flattened patches and the vector of position information are added for a serialized representation of the global information of the image.

#### 3.4.2. Pre-training with MAE

**Figure 5:**
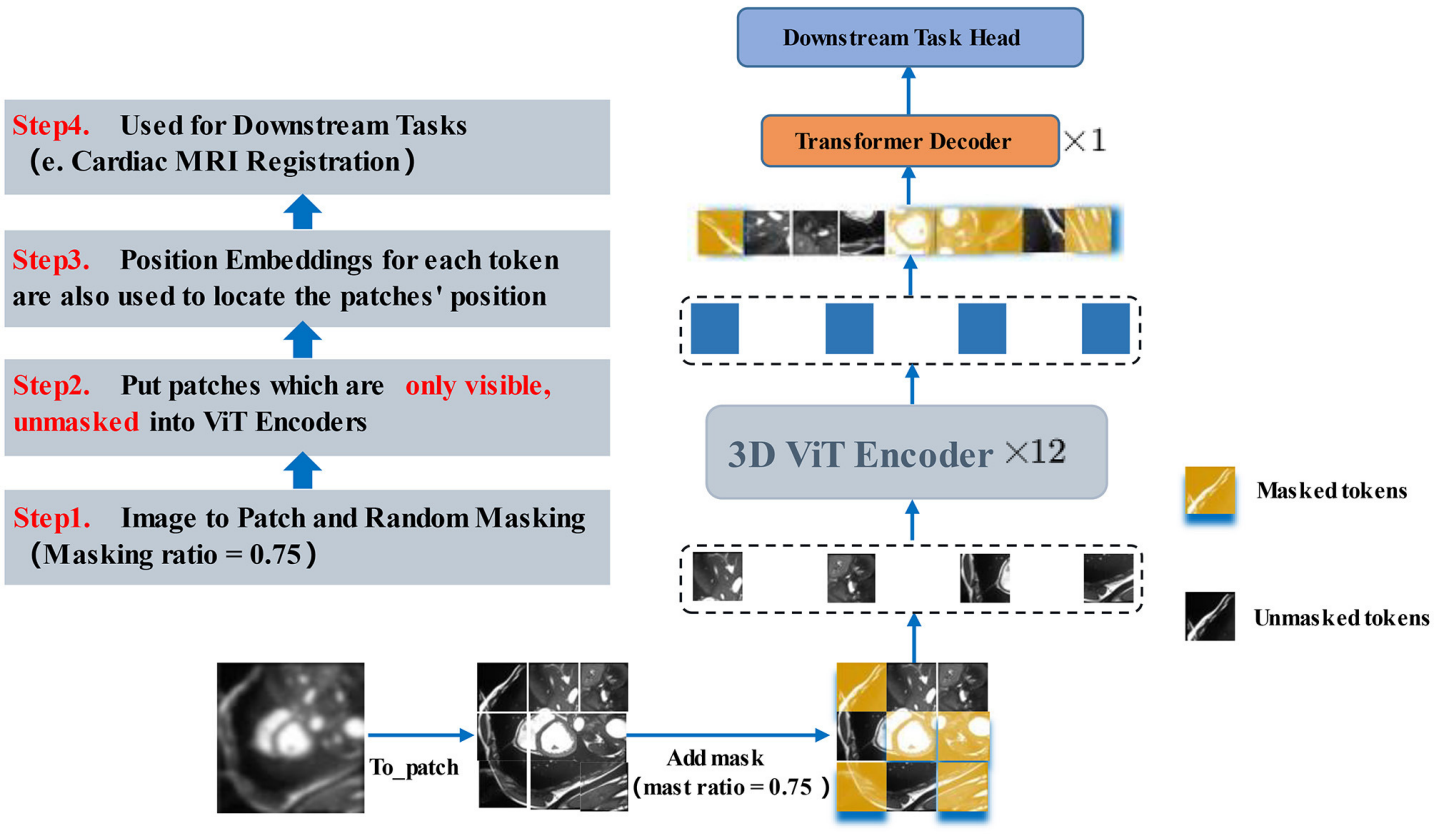
3D MAE Transformer. After the masked operation, only the unmasked patches are fed into ViT. After the linear mapping into one-dimensional tokens, the blank positions (yellow parts) are all filled by the same vector of the same dimension and then decoded by a layer of Transformer decoder. The next layer will be fine-tuned in accordance with the downstream task.

The core part of the proposed method introduces a self-supervised learning strategy by designing 3D ViT as an autoencoder structure with random masked operations to allow the encoder to learn more high-level abstract features and by employing an asymmetric encoder -decoder structure as the core structure of the registration network. Figure 5 illustrates the 3D MAE framework adopted. The feature map is sliced into overlap patches (patch size = 8) in the conventional ViT approach, accessing the position embedding. For the above patches, the patches above half of the ratio (masking ratio = 0.75) are masked, only a small portion of patches that are visible to the encoder are kept, and then the patches required to be masked are calculated. Next, random indices are obtained and divided into the masked and unmasked parts. The unmasked part is the shallow representation of the high-level features, while the masked part is represented by a shared and learnable vector. Each masked patche can be represented as the same vector. As depicted in Figure 5, only the unmasked patches are fed into ViT after the masked operation. After the linear projection, the patches are converted to one-dimensional tokens, and the blank positions (yellow parts) are all filled by the same vector of the same dimension and then decoded by a layer of Transformer decoder. In the MAE, the MSE is used as the reconstruction loss function, and the reconstruction effect is measured by obtaining the MSE between the reconstructed image and the original image in the pixel space.

#### 3.4.3. Designed architecture applied to downstream tasks

A simple layer is designed as the registration head according to the downstream registration task. Before this layer, five CNN decoder layers are also designed to reconstruct the feature representation obtained by the Transformer Block. Subsequently, the feature representation is recovered to the image data format and gradually upsampled back to the original resolution, as presented in Figure 5.

### 3.5. Loss functions in the registration model

The loss function in the registration model consists of a mean square error (MSE) similarity loss and a regularized smoothing loss based on a folding penalty and the sum of the two is used as the loss between the moving image *M*, the fixed image *F*, and the deformation field φ. The loss function is given by:


(16) $$\begin{array}{l} L\left(F,M,\varphi \right)=L_{MSE}\left(F,M,\varphi \right)+\alpha P \end{array}$$

where $L_{MSE}\left(F,M,\varphi \right)$ is the mean square error similarity loss, α is the regularization parameter, *P* is the regularization loss based on the folding penalty, and the two parts of the loss function are formulated as:


(17) $$\begin{array}{l} {ℒ}_{MSE}(F,M,\varphi )=ℒ(\Theta )=\frac{1}{\Omega }\sum_{\Theta \in \omega }[F(\Theta )-M{}^{\circ}\varphi (\Theta ){]}^{2} \end{array}$$

(18) $$\begin{array}{l} P=\frac{1}{V}\int_{0}^{X}\int_{0}^{Y}\int_{0}^{Z}[{\left(\frac{\partial^{2}T}{\partial x^{2}}\right)}^{2}+{\left(\frac{\partial^{2}T}{\partial y^{2}}\right)}^{2}+{\left(\frac{\partial^{2}T}{\partial z^{2}}\right)}^{2}\\ \;+2{\left(\frac{\partial^{2}T}{\partial xy}\right)}^{2}+2{\left(\frac{\partial^{2}T}{\partial xz}\right)}^{2}+2{\left(\frac{\partial^{2}T}{\partial yz}\right)}^{2}]dxdydz \end{array}$$

In the mean square error similarity loss function, Θ is the network parameter to be learned, Ω represents the image domain, and *M* ∘ φ(Θ) denotes the moving image after spatial transform (warped layer); in the regularized loss function based on the folding penalty, the essence of the function is to penalize the folding region of the deformation field, *V* is the volume of the 3D image domain, *T* is the local spatial transformation, and adding this term minimizes the second-order derivative of the local transformation of the deformation field, which leads to an affine transformation of the local deformation field and thus enhances the smoothness of the global deformation field.

## 4. Experiments

### 4.1. Preparation of datasets and related setting details

The dataset used for the experiment and the related settings are described. In this study, the dataset applied is the publicly available benchmark dataset from the automated cardiac diagnosis challenge (31) (ACDC) in 2017. This dataset contains short-axis cardiac 3D MR images from a total of 150 cases for two-time frames of initial frame-end frame, and the public dataset applied provides standard segmentation labels for three parts (including the left ventricle (LV), the left ventricular myocardium (Myo), and the right ventricle (RV)) for the registration task, which involves five categories of cases (including normal, heart failure with infarction, dilated cardiomyopathy, hypertrophic cardiomyopathy, and right ventricular abnormalities). Hundred cases of the above 150 cases contain the triple segmentation labels, while 50 cases do not contain labels. The same dataset is employed for the pre-training task and the downstream task. For the pre-training task, 250 cases are employed for training, and 50 cases are applied for validation (only include images). For the downstream task, the image parts are extracted in 40 cases (1–40), and the complete cardiac cycle images of 50 cases (101–150) for a total of 90 cases are extracted as the training set, 20 cases (41–60) containing images and labels are extracted as the validation set, and 40 cases of data from cases (61–100) are extracted as the test set. At the data preprocessing stage, all the images are cropped to 64 × 128 × 128, the random flip is adopted as the data augment method for the training set to increase the sample size of the dataset. Furthermore, the label pixel normalization method is applied for the validation and test sets to preprocess the data.

### 4.2. MAE architecture for pre-training

In the pre-training task, the three variants of MAE architecture (MAE-ViT-Base, MAE-ViT-Large, and MAE-ViT-Huge) are adopted to pre-train the heart dataset to compare how well the model learns cardiac image features. Unlike the original method of pre-training with the ImageNet-1K (32) dataset, the ACDC dataset is employed, which is divided into 250 cases and 50 cases for training and validation, respectively. The learning rate is set to 1e-4, and MAE pre-training is run for 500 epochs. Moreover, the batch size is set to 2, and the masking ratio is set to 0.75 (default setting) to save the pre-trained MAE model obtained in the pre-training stage for testing some subsequent results.

### 4.3. Downstream task–Cardiac MRI registration

The method proposed in this study is a hybrid network of CNN and Transformer structures, while some structures with Transformer structures are employed as the main backbone network to access in the registration task for comparison. Thus, VoxelMorph is adopted as the baseline network. The PyTorch framework is employed to implement all methods for the comparison experiments. The MONAI framework is used to visualize the registration results, and the methods of the experiments are completed on an NVIDIA RTX 3090 GPU. The Adam optimizer and the step decay (power = 0.9) learning rate reduction strategy are employed in all neural networks. nii format is converted into 3D volume npz format for two time frames from the dataset, and the two-time frames in the respective 3D format in the training sets are converted into fixed image and moving image, respectively. Subsequently, the validation sets and test sets let the image and label of each of the two-time frames form a 3D image pair. On that basis, the respective image is matched with the image of another time frame in a random combination, thus forming four pairs of fixed image and moving image (360 pairs, 80 pairs, and 160 pairs). The proposed framework is compared primarily with several typical methods based on deep learning, which include the baseline framework for registration, VoxelMorph, and three networks [CoTr-based (33) registration network, PVT-based (34) registration network, and ViT-V-Net)] for several applications of the Transformer backbone. The single-channel fixed image and the moving image are combined into a 3D grayscale image with a channel number of 2 as the input of the network. All inputs are subjected to the same preprocessing. The batch size is set to 2, the initial learning rate is set to 0.0001, and the training rounds of 500 epochs are set. The whole process is performed by downsampling the input five-dimensional tensor. Subsequently, the obtained high-level feature representation is divided into equal-sized patches through patch embedding operation. For patches, the remaining visible unmasked patches are fed into the encoder of the 3D vision transformer, so the deformation is achieved from the input image to the predicted densely aligned deformation field using the spatial transformer network. The proposed model is trained by optimizing the loss function for the similarity between the fixed and moving images. For the metrics to evaluate the registration effect, dice coefficient (DSC) and hausdorff distance (HD) are selected to evaluate the 3D registration results.

## 5. Results

### 5.1. Cardiac image in MAE reconstruction

**Figure 6:**
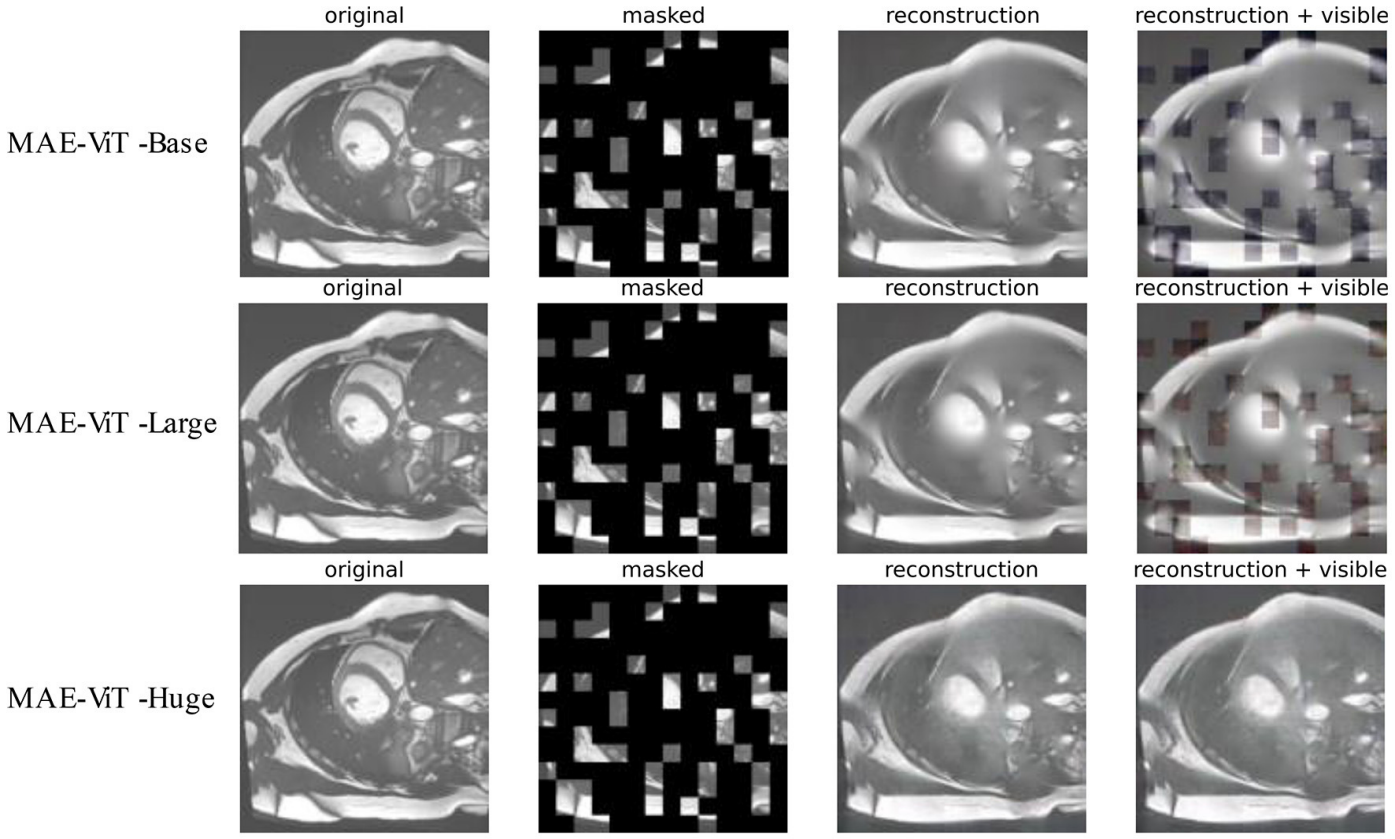
Reconstruction effect of cardiac images on MAE's three variants. The pre-processed original images, masked images, reconstructed images, and MAE reconstruction pasted with visible patches are presented from left to right.

The reconstruction effect for cardiac MRI is tested by pre-training the model on the ACDC dataset. Figure 6 presents the results of three variants of MAE architecture's reconstruction at a mask ratio of 0.75. As revealed by the results, although the resolution of visible patches in the reconstructed image is reduced, and the three model variants differ in their reconstruction of cardiac images. The MAE can still recover the lost information from the pixels around the missing patches effectively. The recovered features can be better applied to downstream tasks.

### 5.2. Cardiac MRI registration

The method applied takes the dice coefficient and hausdorff distance as measurement metrics. The proposed method is compared with several advanced registration methods currently available, and the experiments are performed on 150-cases ACDC dataset. The comparison results achieved for dice performance and Hausdorff distance are listed in Table 1. Some representative registration methods are based on deep learning, including the unsupervised registration baseline -VoxelMorph, as well as the registration network with 3D PVT-based, CoTr-based, and ViT-V-Net.

**Table 1:** Comparison of image registration performance (including dice performance and Hausdorff distance) of five different methods on the ACDC dataset.

**Methods**	**LV**		**Myo**		**RV**		**Avg**	
	**DSC**	**HD**	**DSC**	**HD**	**DSC**	**HD**	**DSC**	**HD**
VoxelMorph	0.847	5.75	0.743	6.23	0.754	9.32	0.781	7.10
CoTr-Based	0.847	5.59	0.776	6.12	0.768	9.25	0.797	6.99
PVT-Based	0.848	5.37	0.745	6.08	0.778	9.12	0.79	6.86
ViT-V-Net	0.856	5.51	0.789	5.96	0.783	8.78	0.809	6.75
**The proposed method**	**0.858**	**5.49**	**0.792**	**5.93**	**0.785**	**8.65**	**0.812**	**6.69**

The best results are achieved and highlighted by the bold values.

**Figure 7:**
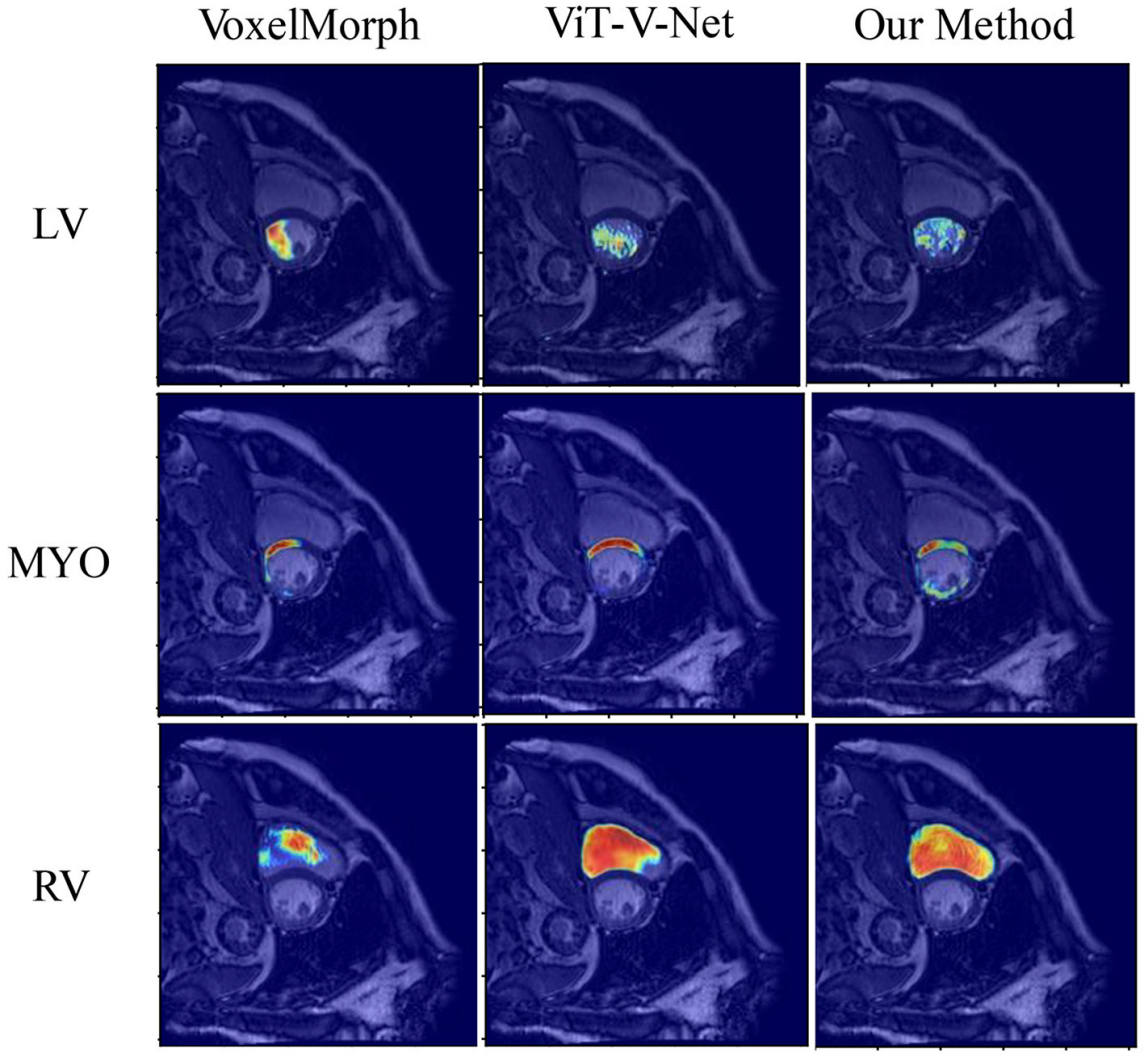
Visualization results of the attention heat map of the ACDC dataset in several models.

The visualization results of the attention heat map of the ACDC dataset in several models are shown in Figure 7. We compared the proposed method with VoxelMorph and ViT-V-Net to compare the models in terms of feature aggregation. In the visualization results, brighter regions indicate a higher degree of feature aggregation. These visualization results of the attention heat map show that all three methods can aggregate different features in three regions of the left and right ventricles and ventricular walls. In contrast, our method can wrap the target contour region more comprehensively.

**Figure 8:**
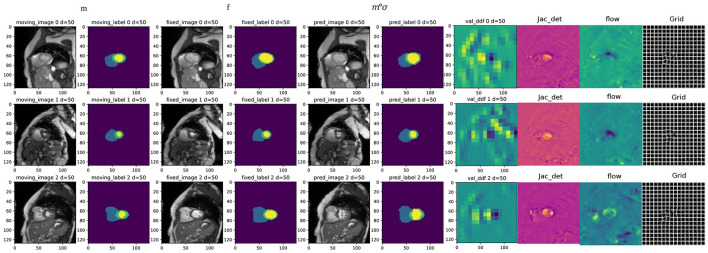
Examples of registration results from the proposed method, columns 1 and 3 are the moving image and fixed image from three different periods; columns 2 and 4 are the triple classification labels for the left ventricle, left ventricular myocardium, and right ventricle; columns 5 and 6 represent the warped original image and the warped image with labels, respectively; column 7 is the dense deformable field generated from fixed image and moving image; column 8 is the visualization result of the Jacobian determinant, as the dense displacement vector field (DVF); columns 9 and 10 are the registration flow filed and displacement field generated from the deformed images.

**Figure 9:**
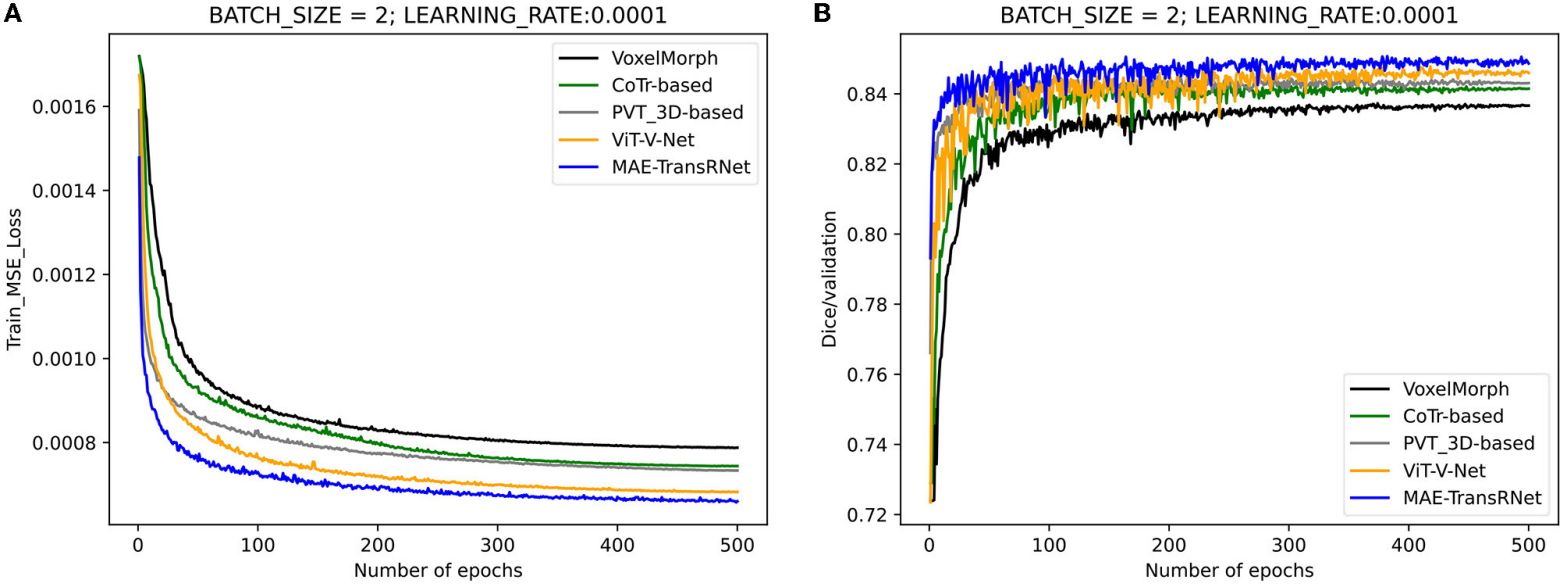
Performance comparison of the training and validation stage of different methods. Compared with other methods, the loss values of the proposed method are kept at a lower level during the training process, and the dice performance values obtained from the validation set are higher. **(A)** Training loss values under the comparison of different registration methods. **(B)** Validation dice performances under the comparison of different registration methods.

**Figure 10:**
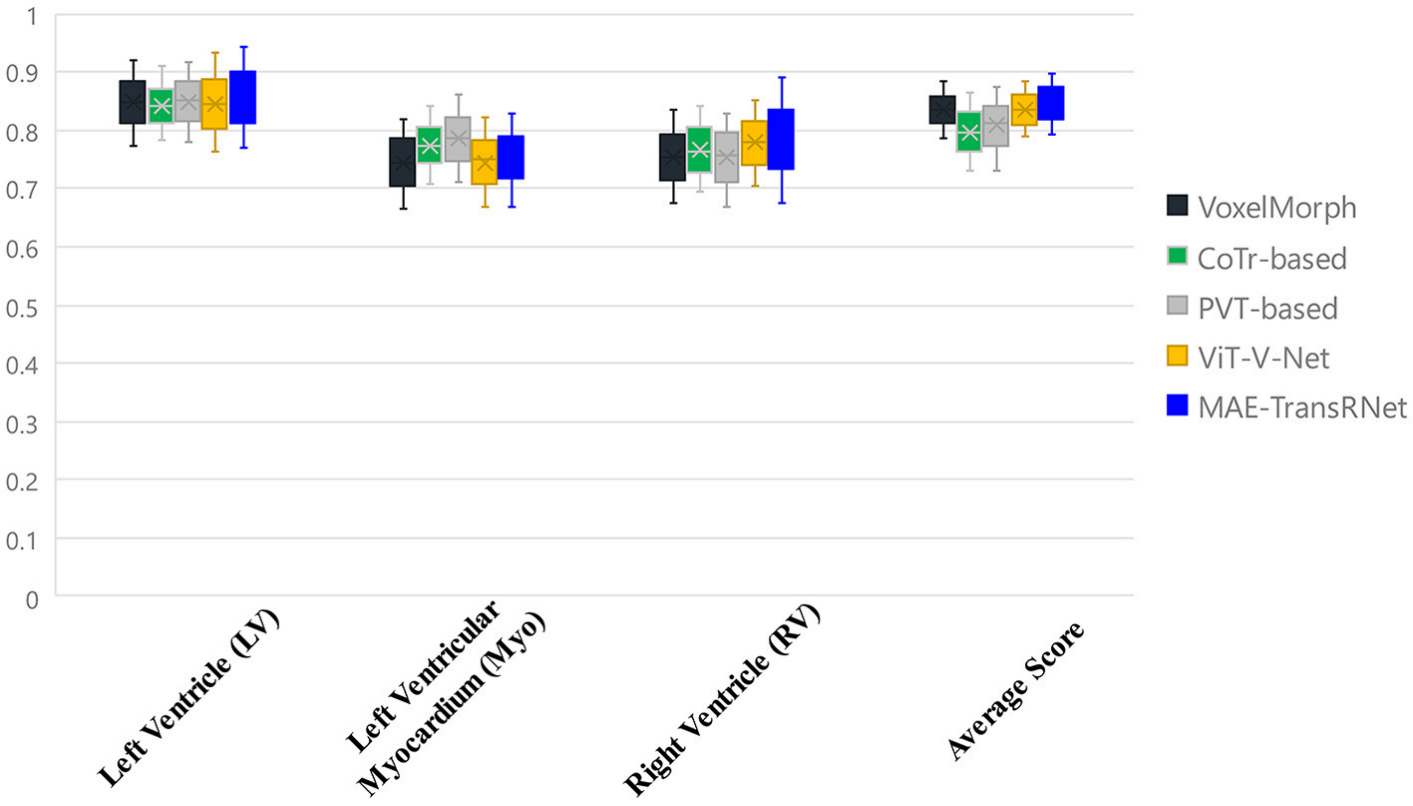
Boxplots to describe the variability of dice performance obtained by different registration methods for the same anatomical structure.

**Figure 11:**
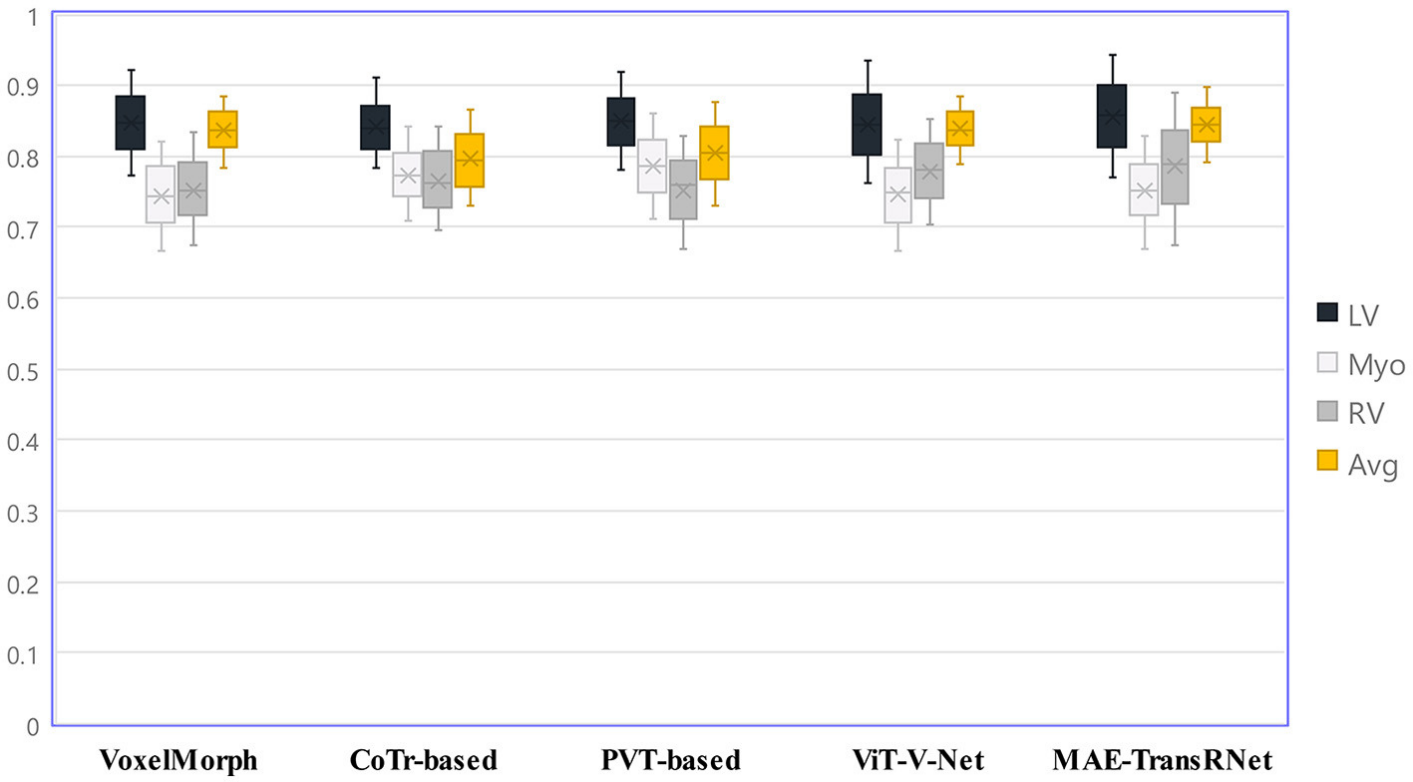
Boxplots to describe the variability of dice performance for various anatomical structures obtained under the same registration method.

Figure 8 presents the registration results of the whole cardiac organ and the left and right ventricles obtained by the proposed method and the generated deformation fields, including three different periods of registration. The proposed method is capable of enhancing the dice performance by nearly 0.01 and decreasing the Hausdorff distance by about 0.1, respectively, compared with other methods, and the loss values of the proposed method are kept at a lower level during the training process, and the dice performance values obtained from the validation set are higher (Figures 9A, B). In the meantime, we set the value of contextual neighbors in self-attention to 100 by default and compare the time complexity of traditional self-attention, naive local intensive attention, and hybrid local dense attention in our model. The results show that the model can improve model performance while maintaining a low time complexity. These results are shown in Table 2. In brief, the MAE-TransRNet achieves better registration results and verifies the effectiveness of MAE, SE, and HyMHSA modules introduced into the registration task. In the meantime, we used boxplots to describe the variability of dice performance obtained by different registration methods for the same anatomical structure and also the variability of dice performance for various anatomical structures obtained using the same registration method (Figures 10, 11).

**Table 2:** Comparison of proposed methods with different attention mechanisms including time complexity and registration performance.

**Method**	**Complexity**	**DSC Avg**	**HD Avg**
MHSA	$O\left(N^{2}D\right)$	0.807	6.81
LDSA	O(Ncn)	0.801	6.71
HyMHSA	O(N(N+cn))	0.812	6.69

*N* is the length of the input feature and *cn* is the context neighbor's value.

## 6. Ablation study

To evaluate the effect of our proposed MAE-TransRNet more accurately, a series of ablation experiments are set to verify the performance of the model under different settings, including the masking ratio size, and whether to add different dimensions to the Transformer encoder and CNN modules, and the effect of the MAE model size on the effect of the registration task. All the training epoch is set to 500.

### 6.1. Masking ratio

The default value of the masking ratio is set to 0.75 as the baseline framework for the experiment, and the experimental settings are used when the masking ratio is set to other values to explore the effect of the masking ratio on the final registration effect. The result is listed in Table 3.

**Table 3:** Ablation study about different masking ratios in the ACDC dataset for registration.

**Masking ratio**	**LV**		**Myo**		**RV**		**Avg**	
	**DSC**	**HD**	**DSC**	**HD**	**DSC**	**HD**	**DSC**	**HD**
0.85	0.854	5.52	0.773	6.12	0.783	8.75	0.803	6.8
**0.75**	**0.858**	**5.49**	**0.792**	**5.93**	**0.785**	**8.65**	**0.812**	**6.69**
0.65	0.857	5.42	0.776	5.89	0.783	8.62	0.812	6.64
0.375	0.858	5.42	0.794	5.85	0.786	8.62	0.813	6.63
**0.125**	**0.859**	**5.39**	**0.795**	**5.78**	**0.788**	**8.58**	**0.814**	**6.58**

The best results are achieved and highlighted by the bold values.

### 6.2. SE module's position

The SE module is introduced in the MAE-TransRNet architecture. Because of the Transformer-ConvNet architecture, the SE module is embedded into the Transformer block and the scSE module into the CNN block, and the effect of SE embedding on the model is compared at the above two positions. It is found that the dice coefficients and HD of the registration are slightly improved by introducing SE module either in the Transformer block or in the CNN block, and the results are better when SE module is introduced in both parts of the architecture, thus suggesting that the attention mechanism based on the channel and spatial dimensions in the Transformer block and CNN block is beneficial. The results of our experiments are listed in Table 4.

**Table 4:** Ablation experiments on the location of SE module embedding.

**SE module position**	**LV**		**Myo**		**RV**		**Avg**	
	**DSC**	**HD**	**DSC**	**HD**	**DSC**	**HD**	**DSC**	**HD**
Transformer	0.856	5.51	0.789	5.98	0.783	8.68	0.811	6.72
CNN Block	0.854	5.65	0.793	6.07	0.782	8.73	0.809	6.82
**Trans+CNN**	**0.858**	**5.49**	**0.792**	**5.93**	**0.785**	**8.65**	**0.812**	**6.69**

The best results are achieved and highlighted by the bold values.

### 6.3. Model scaling

**Figure 12:**
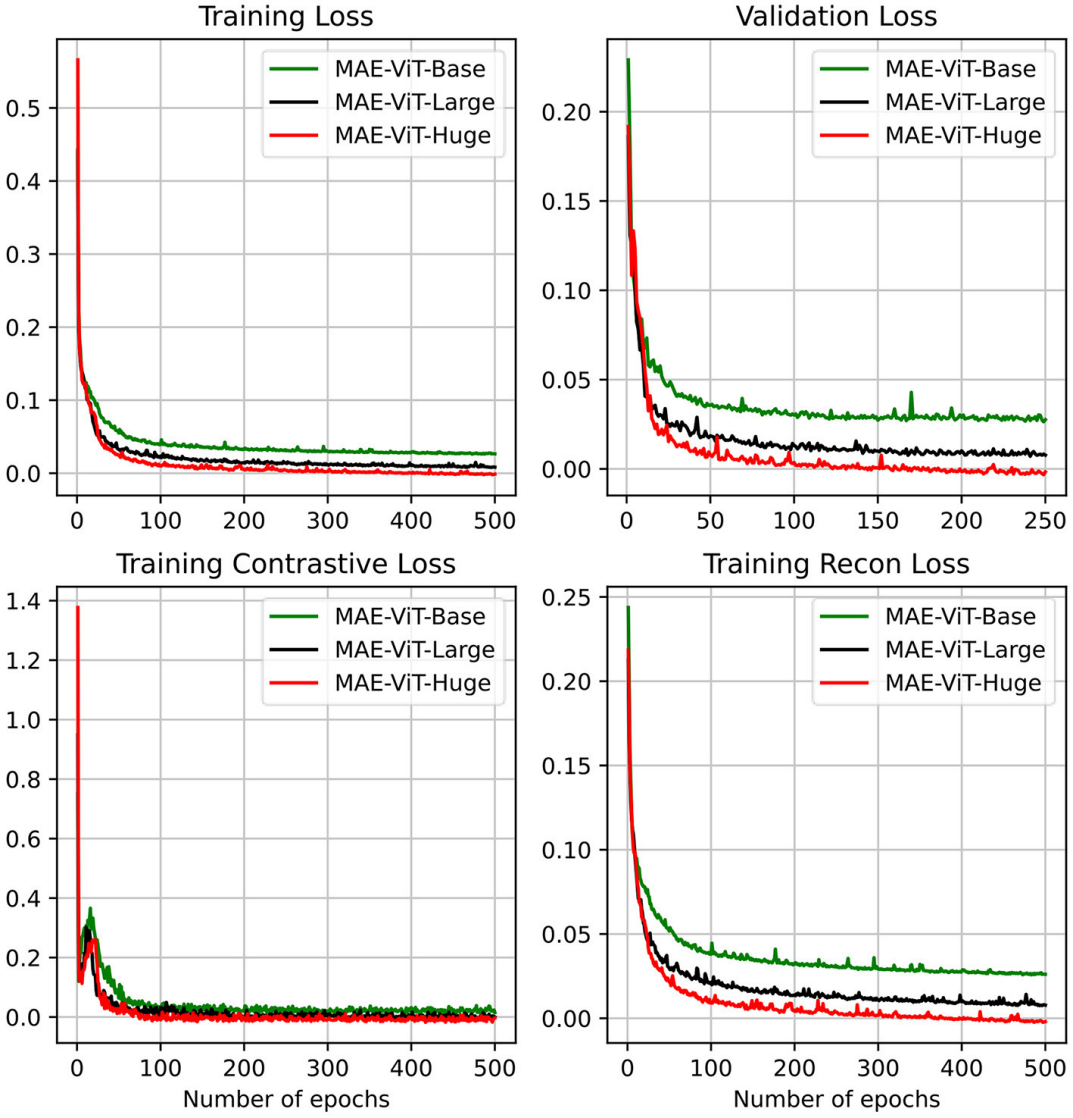
MAE's pre-training result on the ACDC dataset, including training loss, validation loss, training contrastive loss, and training reconstruction loss. We consider three different MAE variants.

Finally, we provide an ablation study on different model sizes of MAE pre-training model. In particular, three different configurations, including the “Base,” “Large,” and “Huge” models, are investigated. For the “base” model, the patch size, encoder dim, MLP dim, number of ViT layers, and number of ViT heads are set to be 16, 768, 3,072, 12, and 12. It is concluded that larger model results in a better performance. For the huge computation cost, the MAE-ViT-Base model is applied to all the experiments. The result and the related configuration are shown in Tables 5, 6. Moreover, the related train loss value is presented in Figure 12.

**Table 5:** Comparison of image registration performance in three variants of MAE pre-training model (including dice performance and Hausdorff distance) on the ACDC dataset.

**Pretrain-Model**	**LV**		**Myo**		**RV**		**Avg**	
	**DSC**	**HD**	**DSC**	**HD**	**DSC**	**HD**	**DSC**	**HD**
Base	0.858	5.49	0.792	5.93	0.785	8.65	0.812	6.69
Large	0.859	5.47	0.795	5.96	0.786	8.6	0.813	6.68
Huge	0.861	5.42	0.794	5.87	0.786	8.37	0.814	6.56

**Table 6:** Three variants of MAE's detail about configuration and parameters.

**Model**	**Patch size**	**Encoder dim**	**Mlp dim**	**ViT layers**	**ViT head**	**Params**
Base	16	768	3,072	12	12	63.837M
Large	16	1,024	4,096	24	16	244.455M
Huge	14	1,280	5,120	32	16	387.248M

## 7. Discussion

An unsupervised learning deformable image registration model is proposed based on Transformer-ConvNet. It has been implemented to predict the spatial transformation parameters between input image pairs by introducing ViT. There are two differences between most deep learning-based methods, especially some methods that introduce the Transformer as follows:

(1) The proposed model is trained by continuously optimizing the image similarity metric without any label as ground truth, while the label is used to support validation and testing. Thus, the registration effectiveness is measured.

(2) We designed the core of the Transformer as a self-encoder and lightweight decoder structure with a MAE, turning the feature extraction prior to the registration downstream task into a self-supervised learning task.

The cardiac MRI dataset for the ACDC is evaluated. The experimental results suggest that the model can outperform the baseline model of deep learning-based deformable registration and slightly outperform some other Transformer-based registration methods. A MAE is applied to the heart registration task first from the difference between text and image information. The method of masking more than half of the patches significantly reduces the redundancy of images, making the feature extraction task more challenging and forcing the model to learn more deeply hidden and better representations. Our purpose in introducing two SE modules is to enhance the feature representation capability of the Transformer structure and the CNN structure. The purpose of introducing the scSE module in the CNN structure is to help us dig deeper into the fine-grained information of the feature map by considering the importance of features in the channel and spatial dimensions to the fine-grained pixel information in the heart image; we introduce the SE module in the self-focus mechanism, hoping to analogize the application scenario of SE in convolution to do query, key, and value in the self-focus computation, respectively. We successfully introduce some convolutional induction bias in the Transformer module to enhance the extraction of local information. Also, we are the first to use this kind of local dense attention in the vision domain, especially in the alignment task. We believe that this self-attention mechanism based on local neighbor context is useful for medical image analysis tasks. The results of several comparison experiments and ablation studies suggest that using the MAE for medical image registration tasks is of great significance in the effect improvement, and the MAE with different scales has a slight difference in the reconstruction effect of cardiac images. It is more appropriate to select “Base” as the baseline model to avoid a high cost of computation. It is worth discussing that, unlike the results when the MAE with a high masking ratio is applied to natural images (e.g., ImageNet-1K dataset), a high masking ratio does not make the MAE achieve the optimal result in medical image tasks. Since the masking ratio is continuously adjusted downward, the effectiveness of our registration tasks is increased slightly, which also suggests that the masking ratio of MAE has different effects on different image analysis tasks. Moreover, the embedding of the SE module in Transformer-ConvNet structure plays a positive role in feature extraction to a certain extent.

However, the effect of the proposed method compared with other methods on the cardiac registration task does not differ significantly between models, probably because the dataset size is relatively small and the model parameters are great. In addition, for the part of MAE, before feeding into the decoder, a part of the token in the blank position is filled in by sharing the learnable vector, which essentially generates non-existent content and is easy to mislead the original features of the image. Accordingly, if the potential impact is further considered, our future research is devoted to the design of the model to be more lightweight, considering the realism of the underlying information representation, while trying to scale up a certain amount of dataset size to further enhance the registration performance.

## 8. Conclusion

An unsupervised learning deformable image registration method is proposed based on Transformer-ConvNet structure, which changes the original ViT structure, introduces mask operations, and does not require segmentation labels as registration information. Furthermore, we introduce a new multi-head self-attention mechanism that sets the range of the model considering neighbors so that the attention module only computes contextual information within a limited distance from the current location. The result of this study verifies that the MAE-TransRNet can achieve results comparable to several popular methods at present and still has much room for improvement. Future research may be extended to multimodal cardiac image registration tasks.

## Data availability statement

Publicly available datasets were analyzed in this study. This data can be found at: https://www.creatis.insa-lyon.fr/Challenge/acdc/index.html. Codes and models are available at: https://github.com/XinXiao101/MAE-TransRNet.

## Author contributions

XX, SD, and ZQ conceived this study. YY and YL were the developers of computer-aided diagnosis methods. XX and GY completed the data analysis. XX and SD drafted the manuscript. All authors were involved in the finalization of the manuscript and approved the manuscript.
